# Vaccine efficacy against persistent human papillomavirus (HPV) 16/18 infection at 10 years after one, two, and three doses of quadrivalent HPV vaccine in girls in India: a multicentre, prospective, cohort study

**DOI:** 10.1016/S1470-2045(21)00453-8

**Published:** 2021-11

**Authors:** Partha Basu, Sylla G Malvi, Smita Joshi, Neerja Bhatla, Richard Muwonge, Eric Lucas, Yogesh Verma, Pulikkottil O Esmy, Usha Rani Reddy Poli, Anand Shah, Eric Zomawia, Sharmila Pimple, Kasturi Jayant, Sanjay Hingmire, Aruna Chiwate, Uma Divate, Shachi Vashist, Gauravi Mishra, Radhika Jadhav, Maqsood Siddiqi, Subha Sankaran, Priya Ramesh Prabhu, Thiraviam Pillai Rameshwari Ammal Kannan, Rintu Varghese, Surendra S Shastri, Devasena Anantharaman, Tarik Gheit, Massimo Tommasino, Catherine Sauvaget, M Radhakrishna Pillai, Rengaswamy Sankaranarayanan

**Affiliations:** a Early Detection, Prevention and Infections Branch, International Agency for Research on Cancer, Lyon, France; b Tata Memorial Centre Rural Cancer Project, Nargis Dutt Memorial Cancer Hospital, Barshi District Solapur, Maharashtra, India; c Jehangir Clinical Development Centre, Jehangir Hospital Premises, Pune, India; d Department of Obstetrics and Gynaecology, All India Institute of Medical Sciences, New Delhi, India; e Sikkim Manipal Institute of Medical Sciences, Sikkim Manipal University, Gangtok, Sikkim, India; f Christian Fellowship Community Health Centre, Ambillikai, Dindigul District, Tamil Nadu, India; g Public Health Foundation of India, IIPH-Hyderabad, Hyderabad, India; h Department of Community Oncology, Gujarat Cancer and Research Institute, M P Shah Cancer Hospital, Civil Hospital Campus, Asarwa, Ahmedabad, India; i Civil Hospital, Aizawl, Mizoram, India; j Department of Preventive Oncology, Centre for Cancer Epidemiology, Tata Memorial Centre, Homi Bhabha National Institute, Parel, Mumbai, India; k Cancer Foundation of India, Kolkata, West Bengal, India; l Rajiv Gandhi Centre for Biotechnology, Poojappura, Thiruvananthapuram, Kerala, India; m Human Biology Division, Fred Hutchinson Cancer Research Centre, Seattle, WA, USA; n Department of Health Disparities Research, Division of Cancer Prevention and Population Sciences, University of Texas MD Anderson Cancer Center, Houston, TX, USA; o Research Triangle Institute International India, New Delhi, India

## Abstract

**Background:**

A randomised trial designed to compare three and two doses of quadrivalent human papillomavirus (HPV) vaccine in adolescent girls in India was converted to a cohort study after suspension of HPV vaccination in trials by the Indian Government. In this Article, the revised aim of the cohort study was to compare vaccine efficacy of single dose to that of three and two doses in protecting against persistent HPV 16 and 18 infection at 10 years post vaccination.

**Methods:**

In the randomised trial, unmarried girls aged 10–18 years were recruited from nine centres across India and randomly assigned to either two doses or three doses of the quadrivalent HPV vaccine (Gardasil [Merck Sharp & Dohme, Whitehouse Station, NJ, USA]; 0·5 mL administered intramuscularly). After suspension of recruitment and vaccination, the study became a longitudinal, prospective cohort study by default, and participants were allocated to four cohorts on the basis of the number vaccine doses received per protocol: the two-dose cohort (received vaccine on days 1 and 180 or later), three-dose cohort (days 1, 60, and 180 or later), two-dose default cohort (days 1 and 60 or later), and the single-dose default cohort. Participants were followed up yearly. Cervical specimens were collected from participants 18 months after marriage or 6 months after first childbirth, whichever was earlier, to assess incident and persistent HPV infections. Married participants were screened for cervical cancer as they reached 25 years of age. Unvaccinated women age-matched to the married vaccinated participants were recruited to serve as controls. Vaccine efficacy against persistent HPV 16 and 18 infections (the primary endpoint) was analysed for single-dose recipients and compared with that in two-dose and three-dose recipients after adjusting for imbalance in the distribution of potential confounders between the unvaccinated and vaccinated cohorts. This trial is registered with ISRCTN, ISRCTN98283094, and ClinicalTrials.gov, NCT00923702.

**Findings:**

Vaccinated participants were recruited between Sept 1, 2009, and April 8, 2010 (date of vaccination suspension), and followed up over a median duration of 9·0 years (IQR 8·2–9·6). 4348 participants had three doses, 4980 had two doses (0 and 6 months), and 4949 had a single dose. Vaccine efficacy against persistent HPV 16 and 18 infection among participants evaluable for the endpoint was 95·4% (95% CI 85·0–99·9) in the single-dose default cohort (2135 women assessed), 93·1% (77·3–99·8) in the two-dose cohort (1452 women assessed), and 93·3% (77·5–99·7) in three-dose recipients (1460 women assessed).

**Interpretation:**

A single dose of HPV vaccine provides similar protection against persistent infection from HPV 16 and 18, the genotypes responsible for nearly 70% of cervical cancers, to that provided by two or three doses.

**Funding:**

Bill & Melinda Gates Foundation.

## Introduction

A combined strategy of high-coverage human papillomavirus (HPV) vaccination of girls aged 9–14 years, twice-lifetime screening of women at 35 years and 45 years of age, and effective treatment of those with cervical neoplasia can potentially eliminate cervical cancer as a public health problem.^1^ The inability of nearly two-thirds of low-income and lower-middle-income countries to introduce the HPV vaccine into their national immunisation programme is a major stumbling block for global elimination of the disease.^2^ These countries have a disproportionately high burden of cervical cancer, but not enough resources to afford the HPV vaccine, which is currently recommended to be administered with at least two doses. WHO estimated the global coverage of the HPV vaccine to be only 15% in 2019.^3^ The fragile health systems of low-income and middle-income countries (LMICs) are inadequately prepared to face the formidable challenge of delivering two doses of the vaccine to adolescent girls.^4^ These countries will be further affected by the huge gap in supply and demand, as the global requirement of the vaccine is expected to rise to 120 million doses per year by 2030.^5^, ^6^

**Research in context**

**Evidence before this study**

We searched PubMed between Feb 1 and March 31, 2021, for all types of publications (original articles and reviews) that reported on the efficacy of a single dose of human papillomavirus (HPV) vaccines, without date or language restrictions. We used the terms “HPV vaccine” AND “efficacy” AND “one-dose”. This search retrieved 21 results. The articles were related to several topics: efficacy, effectiveness, immunogenicity, and durability of vaccination schedules of fewer than three doses. The efficacy of a single dose was compared with no vaccination, or with efficacy of two or three doses. The vaccines were either the bivalent or the quadrivalent HPV vaccines. The outcomes included vaccine efficacy on cervical incident HPV infection, on prevalent infection, and on persistent infection. We identified a recent systematic review reporting on the efficacy and immunogenicity of a single dose of an HPV vaccine. The review included three major HPV vaccine trials: the Costa Rica Vaccine trial, the Papilloma Trial Against Cancer in Young Adults trial, and the International Agency for Research on Cancer India HPV vaccine trial. The results of this systematic review as well as other studies suggested that one dose could be as efficacious as two or three doses in healthy women. The authors of the systematic review highlighted the small sample size of the existing studies and their other weaknesses, and acknowledged the need for additional evidence to confirm the preliminary results of the three trials.

**Added value of this study**

We report similar vaccine efficacy of a single dose of quadrivalent vaccine in adolescent girls compared with that of two or three doses at 10 years post vaccination against persistent and incident HPV 16 and 18 infections as well as cervical neoplasia. The major strengths of the study are large number of single-dose recipients, presence of two-dose and three-dose recipients belonging to the same age group as the comparators, and systematic follow-up over many years. The evidence from our study demonstrating high efficacy of single dose of quadrivalent vaccine in prevention of persistent infection from HPV 16, 18, or both helps to address the existing evidence gap identified by the systematic review.

**Implications of all the available evidence**

The evidence generated by our study is consistent with the high efficacy of a single dose of HPV vaccine observed in other clinical trials and in ecological studies nested in national immunisation programmes. More than 90% efficacy of a single dose in preventing persistent HPV 16 and 18 infections indicate that the quadrivalent vaccine (with its limited cross-protection) could potentially prevent around 70% of the cervical cancers—a huge gain for low-income and middle-income countries considering the great benefits of a single dose in reducing programme costs and complexities.

Adoption of a single-dose schedule for the HPV vaccine could potentially address many of the challenges by improving affordability, making the vaccination programmes logistically simpler and more resilient, and helping to tide over the supply crisis. We aimed to describe the efficacy of a single dose of the quadrivalent HPV vaccine compared with two or three doses to protect against incident and persistent cervical HPV infections 10 years post vaccination. We also report the early evidence of protection against cervical precancers among recipients of different vaccine dosing regimens.

## Methods

### Study design and participants

**Figure 1:**
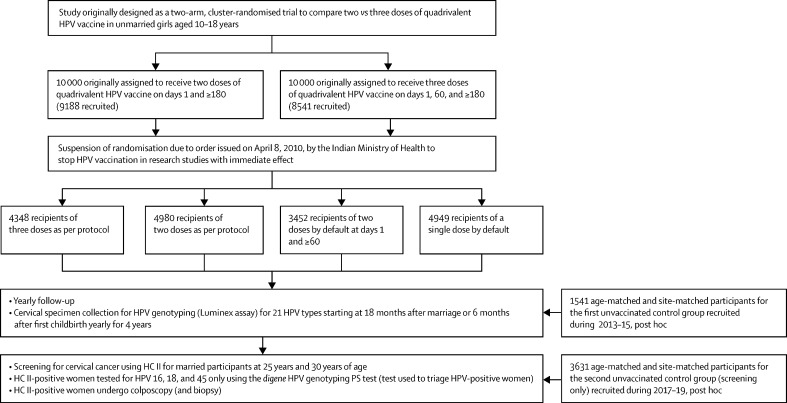
Study flow chart HC II=Hybrid Capture II. HPV=human papillomavirus.

A multicentre, cluster-randomised trial designed to compare the efficacy of two versus three doses of quadrivalent HPV vaccine among unmarried girls aged 10–18 years in India initiated recruitment on Sept 1, 2009. The methods of the study are described elsewhere in detail and are summarised in figure 1.^7^ Briefly, 188 geographical clusters were identified at nine locations spread across different regions of India (appendix p 8), and all eligible girls residing in an individual cluster were enumerated. Using a computer-generated random allocation, the identified clusters at each site were randomly assigned to either a two-dose or three-dose group. The plan was to recruit 10 000 girls in each dose group from the enumerated girls. In response to seven vaccine-unrelated deaths reported in another ongoing HPV vaccination demonstration programme in the country, the Indian Government issued a notification on April 8, 2010, to stop further recruitment and HPV vaccination in all clinical trials.^8^ Our study also stopped recruitment on that date. Until suspension, 17 729 eligible girls from 178 clusters were recruited across nine study sites and had received at least one dose of the vaccine.

After suspension of recruitment and vaccination, the trial was converted to a longitudinal, prospective, cohort study by default. For the cohort study, we recruited two cohorts of unvaccinated women at different time periods to serve as age-and-site-matched controls post hoc.

The study was approved by the ethics committees at the International Agency for Research on Cancer (IARC; Lyon, France) and all collaborating institutions. Every participant signed an informed consent or an assent (if aged <18 years). Either of the parents gave written consent for the girls aged younger than 18 years. The study was monitored by a data safety monitoring board.

### Procedures

Each participant received 0·5 mL of the quadrivalent HPV vaccine (Gardasil; Merck Sharp & Dohme, Whitehouse Station, NJ, USA) administered intramuscularly. After suspension of recruitment and vaccination, participants were distributed across four cohorts on the basis of the doses received per protocol: recipients of three doses on days 1, 60, and 180 or later (three-dose cohort), recipients of two doses on days 1 and 180 or later (two-dose cohort), recipients of two doses on days 1 and 60 or later (two-dose default cohort), and recipients of a single dose of the vaccine (single-dose default cohort).^7^, ^9^

All participants are being followed up yearly, and the follow-up will continue for at least another 5 years to August, 2026. History of receiving an HPV vaccine outside the study was obtained at each visit along with routine medical history; any participants receiving HPV vaccine outside the study were excluded from further follow-ups. We collected information on clinically significant medical events at each visit. However, the data were not analysed for this study. Participants became eligible to provide cervical specimens for individual detection of any of the 21 HPV genotypes 18 months after marriage or 6 months after first childbirth, whichever was earlier. One cervical specimen was collected per year (within a window of minus 2 months and plus 6 months) from eligible participants for 4 consecutive years. Participants in whom a new HPV infection was detected in the fourth sample were advised to return to provide a fifth sample after 1 year. Genotyping was done with the Luminex assay (Luminex, Austin**,** TX, USA).

Participants were invited to cervical cancer screening once they reached 25 years of age and were married. A well validated HPV detection test (Hybrid Capture II [HC-II]; Qiagen, Gaithersburg, MD, USA) was used as the screening test, for which cervical specimens were collected by a trained provider. Positive specimens were further tested with the *digene* HPV genotyping PS test (Qiagen) for the qualitative detection of high-risk HPV types 16, 18, and 45. The *digene* PS test detects the genotype associated with cervical neoplasia (if detected) as some of the screening-eligible women might have completed providing samples for Luminex genotyping years before screening. We recalled every HC-II-positive participant for colposcopy. A Swede score of five or more on colposcopy was an indication for obtaining punch biopsies from the areas of worst abnormalities.^10^ Test-positive women not requiring biopsy were recalled after a year for a repeat HC-II test.

The first cohort of unvaccinated married women matched by age and study site to the vaccinated women eligible to provide specimens for genotyping was recruited in 2013–15. The participants belonging to the first unvaccinated cohort provided specimens for Luminex genotyping and were also screened for cervical cancer following the same protocol applicable for vaccinated participants. A second unvaccinated cohort of married women, age and site matched to the screening-eligible vaccinated women, was recruited in 2017–19. Women recruited to the second cohort were only screened for cervical cancer (screening-only cohort) following the protocol described earlier. No specimens were collected from them for the Luminex assay.

Luminex is a type-specific E7 PCR multiplex assay to detect 19 high-risk or probable high-risk types (HPV 16, 18, 26, 31, 33, 35, 39, 45, 51, 52, 53, 56, 58, 59, 66, 68a, 68b, 70, 73, and 82), and two low-risk types (HPV 6 and 11). A centralised facility has been set up for the assay at Rajiv Gandhi Centre for Biotechnology in Thiruvananthapuram (Kerala, India). The laboratory was certified as proficient by the International HPV Reference Centre at the Karolinska Institute in Stockholm (Sweden) following a quality control exercise based on the global HPV DNA proficiency panel 2017.^11^ External quality control of the assay is performed by periodic blinded analysis of a subset of positive and negative specimens at the IARC laboratory in Lyon (France).

HC-II is a semi-quantitative chemiluminescence assay to detect any of the 13 most oncogenic HPV types in the specimen. The specimen should have at least 5000 copy numbers of viral DNA of the targeted HPV types to test positive. The HC-II test is centralised at one of the study sites (Nargis Dutt Memorial Cancer Hospital, Maharashtra, India). All laboratory staff and colposcopists were masked to the vaccination status of participants.

The formalin-fixed cervical biopsy specimens were processed for histopathology at respective study sites and examined by the site pathologists. A trained and experienced gynaecological pathologist examined all the slides centrally at Regional Cancer Center in Thiruvananthapuram. She was masked to the group allocation, colposcopy interpretation, and site histopathology result. Diagnosis made by the expert pathologist was considered as final.

### Outcomes

The primary endpoint of the original randomised, controlled trial aiming to compare two versus three doses of the vaccine was detection of cervical intraepithelial neoplasia grade 2 or worse (CIN2+) lesions, which was the standard endpoint being used by all other vaccine trials at that time. Subsequently, prevention of persistent oncogenic HPV infection was well accepted as a valid surrogate to demonstrate protection against CIN2+ lesions. In this study, we assessed persistent infection from HPV 16, 18, or both (hereafter referred to as HPV 16/18) as the primary outcome. The secondary outcomes were incident HPV 16/18 infections, and HPV 16/18-related CIN2+ lesions. Other exploratory outcomes were persistence and incidence of any one or more of HPV 6 and 11; any one or more of HPV 31, 33, and 45; and any HPV type.

For any participant, incident infection was defined as detection of an HPV type in any one sample, and persistent infection was defined as detection of the same HPV type in two consecutive samples taken at least 10 months apart. Only Luminex assay outcomes were used to assess incidence and persistence. Each enrolled participant contributed once to persistent infections, once to incident infections, or both.

### Statistical analysis

Participants' baseline characteristics were stratified by the vaccine dose received and are presented as proportions. For the incident HPV infection outcomes, the analyses included participants who provided at least one cervical cell sample, and persistent HPV infection analyses included participants with at least two cervical cell sample collections. HPV incidence and persistence outcomes were also stratified by the vaccine dose received and are presented as proportions with their 95% CIs.

Vaccine efficacy was analysed against incident and persistent infections. Vaccine efficacy was calculated as one minus HPV infection rate in the vaccinated group divided by HPV infections rate in the unvaccinated group. The 95% CI for vaccine efficacy was obtained using a two-step approach: first, estimating the proportion of cases in the vaccinated cohort conditioned on the total number of cases in both the vaccinated and unvaccinated cohorts computed using an exact conditional procedure; and, second, using the lower and upper limits of the proportion 95% CI to calculate the 95% CI for vaccine efficacy.^12^, ^13^ To compare vaccine efficacy between the single-dose default cohort and the three-dose and two-dose cohorts, the difference in vaccine efficacy estimates together with their 95% CIs are presented. We excluded the two-dose default group from the efficacy analysis because the schedule was of no practical relevance.

Because of the ad-hoc nature in which the first unvaccinated cohort (the second unvaccinated cohort was not tested for incident and persistent infections) was recruited, several descriptive analyses were done to arrive at an appropriate modification needed to minimise the imbalance in the distribution of potential confounders between the unvaccinated and vaccinated cohorts. To minimise the imbalance, we created strata that would effectively have similar distribution of characteristics, and that, in the absence of vaccination, would have had extremely similar frequencies of HPV infection (appendix p 1). Such strata with similar infection frequencies of the HPV types that are unlikely to be influenced by vaccination were formed using the following steps.

First, we used the non-vaccine targeted HPV types, excluding 31, 33, and 45, as the outcome for HPV types that are unlikely to be influenced by vaccination. We then performed logistic regression models to obtain odds ratios together with their 95% CI and to show that those non-vaccine-targeted HPV types significantly predicted the vaccine-targeted (HPV 16/18) and cross-protective (HPV 31, 33, and 45) outcomes (appendix p 3).

Second, using only data for the unvaccinated cohort, we ran a multivariate logistic regression model to determine the potential confounders (characteristics) that significantly predict these non-vaccine-targeted HPV types. The potential confounders included were study site groups created based on background site-specific HPV infection profile (for more detail see appendix p 2); birth cohort (those born before 1995 and those born in or after 1995 at recruitment); religion (Hindu and others); total number of pregnancies (none, one, and two or more); age at first cervical cell sample collection (<21 years and ≥21 years); time between dates of marriage and first cervical sample collection (<2 years, 2 to <3 years, and ≥3 years); delayed cervical sample collection (not delayed and at least one delayed; a delay was defined as a gap of 18 months or more between the dates of any consecutive cervical cell sample collection); and number of cervical cell sample collections per participant (one to two and three or more). More details are available in the appendix (p 1). Since the site-specific rates were similar between the vaccinated and unvaccinated groups (appendix p 7), and since the Mumbai site did not have women recruited in the unvaccinated group, we used the estimated rate in the vaccinated group in place of the unvaccinated HPV background rate.

Third, we estimated each participant's disease risk score using another logistic regression model that included the variables that were significantly predicting these other non-vaccine targeted HPV types at the 5% significance level. The coefficients from the regression model were used to assign a score to each risk factor. The risk factor scores were then summed to calculate the participant's total risk score, which was in turn used to obtain the estimates of risk (the probability of being infected with a non-vaccine-targeted HPV excluding types 31, 33, and 45).^14^

As the final step, we created five strata from the fitted scores using the lowest level as the first stratum of individuals with minimal or no risk and the remaining four from quantiles of the scores. As such, we avoided assuming that the risk score and the outcome were related through a particular function. More details are in the appendix (p 1).

Five strata with similar risk profiles were used in the direct standardisation method, with the total vaccinated cohort as the standard population, to obtain the effective number of events and effective population sizes for each dose group. These values in turn were used as the true values in the exact conditional model to obtain adjusted vaccine efficacy estimates.

The analyses for the CIN2+ outcome were based on individuals with HC-II testing results, presented as proportions and compared between the unvaccinated and vaccinated cohorts. Significance was inferred when the p value was less than 0·05. The statistical analyses were carried out in Stata (version 15.1) and Just Another Gibbs Sampler software.

This study is registered with ISRCTN, ISRCTN98283094, and ClinicalTrials.gov, NCT00923702.

### Role of the funding source

The funder of the study had no role in study design, data collection, data analysis, data interpretation, or writing of the report.

## Results

Vaccinated participants were recruited between Sept 1, 2009, and April 8, 2010. The first unvaccinated cohort was recruited between April 29, 2013, and June 16, 2015, and the second between Oct 6, 2017, and June 27, 2019. 4348 participants were included in the three-dose cohort, 4980 in the two-dose cohort, 3452 in the two-dose default cohort, 4949 in the single-dose default cohort, 1541 in the first unvaccinated cohort, and 3631 in the second unvaccinated cohort. No participant reported receiving a HPV vaccine outside our study. The median duration of follow-up was 9·0 years (IQR 8·2–9·6) for the vaccinated participants. The distribution of participants by cohorts varied across the study sites (table 1). Sites initiating recruitment later had a higher proportion of single-dose recipients. The distribution of participants belonging to different cohorts who provided at least one cervical sample for HPV genotyping also varied by the study site (appendix p 4). No cervical specimen was collected from the second unvaccinated cohort for genotyping.

**Table 1:** Baseline characteristics in the vaccinated and unvaccinated cohorts

		**Three-dose cohort**	**Two-dose cohort**	**Two-dose default cohort**	**Single-dose default cohort**	First unvaccinated cohort*	**Second unvaccinated cohort**†
All participants recruited		4348	4980	3452	4949	1541	3631
Study site							
	Ambillikai, Tamilnadu	1446 (33·3%)	1532 (30·8%)	111 (3·2%)	211 (4·3%)	200 (13·0%)	600 (16·5%)
	Barshi, Maharashtra	744 (17·1%)	824 (16·5%)	2699 (78·2%)	2825 (57·1%)	189 (12·3%)	1562 (43·0%)
	New Delhi, Delhi	416 (9·6%)	480 (9·6%)	62 (1·8%)	42 (0·8%)	200 (13·0%)	300 (8·3%)
	Ahmedabad, Gujarat	0	0	0	1011 (20·4%)	50 (3·2%)	100 (2·8%)
	Hyderabad, Telangana	0	0	315 (9·1%)	479 (9·7%)	300 (19·5%)	267 (7·4%)
	Mumbai, Maharashtra	0	490 (9·8%)	0	24 (0·5%)	0	0
	Pune, Maharashtra	1266 (29·1%)	1183 (23·8%)	246 (7·1%)	323 (6·5%)	400 (26·0%)	600 (16·5%)
	Gangtok, Sikkim	233 (5·4%)	230 (4·6%)	13 (0·4%)	24 (0·5%)	102 (6·6%)	102 (2·8%)
	Aizawl, Mizoram	243 (5·6%)	241 (4·8%)	6 (0·2%)	10 (0·2%)	100 (6·5%)	100 (2·8%)
Birth cohort at recruitment							
	<1992	177 (4·1%)	193 (3·9%)	234 (6·8%)	193 (3·9%)	109 (7·1%)	1171 (32·2%)
	1992–93	752 (17·3%)	874 (17·6%)	710 (20·6%)	923 (18·7%)	963 (62·5%)	2228 (61·3%)
	≥1994	3419 (78·6%)	3913 (78·6%)	2508 (72·7%)	3833 (77·4%)	469 (30·4%)	236 (6·5%)
Religion							
	Hindu	3925 (90·3%)	4345 (87·2%)	2961 (85·8%)	4768 (96·3%)	1368 (88·8%)	3197 (88·0%)
	Others	423 (9·7%)	635 (12·8%)	491 (14·2%)	181 (3·7%)	173 (11·2%)	437 (12·0%)
Total number of pregnancies							
	None	2165 (49·8%)	2619 (52·6%)	1113 (32·2%)	1824 (36·9%)	197 (12·8%)	530 (14·6%)
	One	1042 (24·0%)	1204 (24·2%)	787 (22·8%)	1276 (25·8%)	545 (35·4%)	760 (20·9%)
	Two or more	1141 (26·2%)	1157 (23·2%)	1552 (45·0%)	1849 (37·4%)	799 (51·8%)	2345 (64·5%)
Age, years		21 (19–22)	21 (20–23)	20 (19–22)	21 (19–22)	20 (19–21)	..
Participants who provided cervical samples		2275	2430	2314	3155	1486	..
Birth cohort at recruitment							
	<1992	145 (6·4%)	156 (6·4%)	204 (8·8%)	168 (5·3%)	99 (6·7%)	..
	1992–93	538 (23·6%)	638 (26·3%)	580 (25·1%)	725 (23·0%)	924 (62·2%)	..
	≥1994	1592 (70·0%)	1636 (67·3%)	1530 (66·1%)	2262 (71·7%)	463 (31·2%)	..
Religion							
	Hindu	2157 (94·8%)	2275 (93·6%)	2004 (86·6%)	3069 (97·3%)	1315 (88·5%)	..
	Others	118 (5·2%)	155 (6·4%)	310 (13·4%)	86 (2·7%)	171 (11·5%)	..
Total number of pregnancies							
	None	279 (12·3%)	309 (12·7%)	209 (9·0%)	313 (9·9%)	182 (12·2%)	..
	One	897 (39·4%)	1014 (41·7%)	616 (26·6%)	1076 (34·1%)	511 (34·4%)	..
	Two or more	1099 (48·3%)	1107 (45·6%)	1489 (64·3%)	1766 (56·0%)	793 (53·4%)	..

Data are n, n (%), or median (IQR).

* Recruited in 2013–15; provided cervical specimen for HPV genotyping with the Luminex assay for 21 HPV types and additionally formed part of the unvaccinated cohort for cervical cancer screening using the Hybrid Capture II test.

† Recruited in 2017–19; formed the other part of the unvaccinated cohort for cervical cancer screening using the Hybrid Capture II test only; no specimen collected for HPV genotyping.

**Figure 2:**
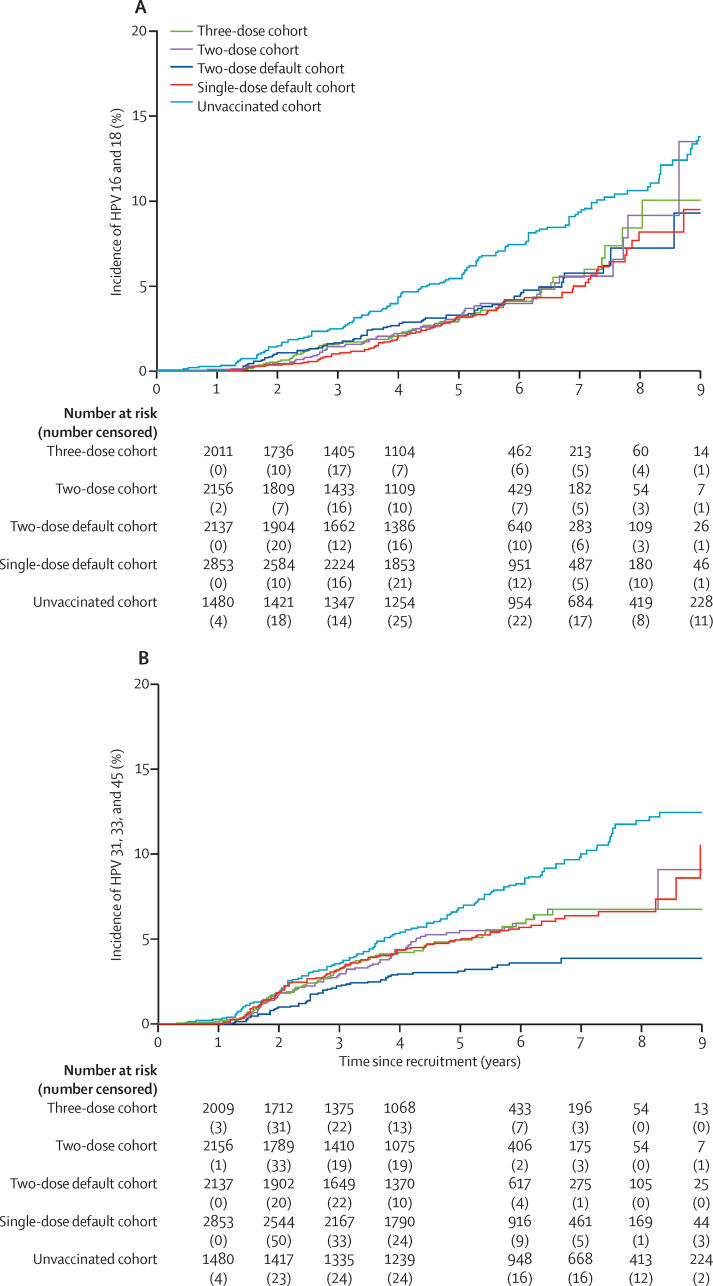
Incidence of HPV 16 and 18 (A), and HPV 31, 33, and 45 (B) HPV=human papillomavirus.

Of the total 17 729 vaccinated women, 10 915 (61·6%) were eligible for HPV genotyping. Among these women, frequency of incident HPV infection was assessed in the 9183 (84·1%) women who provided at least one cervical cell sample (appendix p 5). Incident HPV 16/18 infections were detected in 287 (3·1%; 95% CI 2·8–3·5) of 9183 vaccinated women and 139 (9·4%; 7·9–11·0) of 1484 unvaccinated women (table 2). The frequency of HPV 16/18 incident infections among the single-dose recipients (92 [3·2%; 95% CI 2·6–3·9] of 2858) was similar to that observed in other dose cohorts (table 2; figure 2A). Incident infection from cross-protective HPV types (HPV 31, 33, and 45) was less frequent in vaccinated women than in unvaccinated women, without any difference observed between the three-dose, two-dose, or single-dose default cohorts (table 2, figure 2B). The frequency of incident infection from all vaccine non-targeted types (also excluding cross-protective types) was lower in vaccinated women than in unvaccinated ones. No difference was observed across the vaccinated groups as similar levels of infection were observed across the vaccinated groups.

**Table 2:** Analysis of one-time incident HPV infections and persistent HPV infections in women with at least two samples tested

		**HPV incidence in participants with one or more samples tested**			**HPV infection status in participants with two or more samples tested**		
		Women assessed	Women with incident infections	Proportion of incident infection (95% CI)	Women assessed	Women with persistent infections	Proportion of persistent infection (95% CI)
Women with samples tested		10 667	..	..	7938	..	..
HPV 16 and 18 infections							
	Unvaccinated cohort	1484	139	9·4% (7·9–11·0)	1265	32	2·5% (1·7–3·6)
	Vaccinated cohort	9183	287	3·1% (2·8–3·5)	6673	7	0·1% (0·0–0·2)
	Three-dose cohort	2019	60	3·0% (2·3–3·8)	1460	1	0·1% (0·0–0·4)
	Two-dose cohort	2166	59	2·7% (2·1–3·5)	1452	1	0·1% (0·0–0·4)
	Two-dose default cohort	2140	76	3·6% (2·8–4·4)	1626	4	0·2% (0·1–0·6)
	Single-dose default cohort	2858	92	3·2% (2·6–3·9)	2135	1	0·0% (0·0–0·3)
HPV 6 and 11 infections							
	Unvaccinated cohort	1484	64	4·3% (3·3–5·5)	1265	3	0·2% (0·0–0·7)
	Vaccinated cohort	9183	237	2·6% (2·3–2·9)	6673	4	0·1% (0·0–0·2)
	Three-dose cohort	2019	59	2·9% (2·2–3·8)	1460	1	0·1% (0·0–0·4)
	Two-dose cohort	2166	55	2·5% (1·9–3·3)	1452	0	0·0% (0·0–0·3)
	Two-dose default cohort	2140	55	2·6% (1·9–3·3)	1626	2	0·1% (0·0–0·4)
	Single-dose default cohort	2858	68	2·4% (1·9–3·0)	2135	1	0·0% (0·0–0·3)
Non-vaccine-targeted HPV 31, 33, and 45 infections							
	Unvaccinated cohort	1484	148	10·0% (8·5–11·6)	1265	14	1·1% (0·6–1·8)
	Vaccinated cohort	9183	371	4·0% (3·6–4·5)	6673	34	0·5% (0·4–0·7)
	Three-dose cohort	2019	85	4·2% (3·4–5·2)	1460	7	0·5% (0·2–1·0)
	Two-dose cohort	2166	89	4·1% (3·3–5·0)	1452	11	0·8% (0·4–1·4)
	Two-dose default cohort	2140	61	2·9% (2·2–3·6)	1626	2	0·1% (0·0–0·4)
	Single-dose default cohort	2858	136	4·8% (4·0–5·6)	2135	14	0·7% (0·4–1·1)
Non-vaccine-targeted HPV infections excluding 31, 33, and 45							
	Unvaccinated cohort	1484	403	27·2% (24·9–29·5)	1265	71	5·6% (4·4–7·0)
	Vaccinated cohort	9183	1520	16·6% (15·8–17·3)	6673	211	3·2% (2·8–3·6)
	Three-dose cohort	2019	377	18·7% (17·0–20·4)	1460	49	3·4% (2·5–4·4)
	Two-dose cohort	2166	373	17·2% (15·7–18·9)	1452	47	3·2% (2·4–4·3)
	Two-dose default cohort	2140	293	13·7% (12·3–15·2)	1626	47	2·9% (2·1–3·8)
	Single-dose default cohort	2858	477	16·7% (15·3–18·1)	2135	68	3·2% (2·5–4·0)

HPV=human papillomavirus.

Among the 10 915 vaccinated women eligible to provide cervical specimens, persistent infection was assessed in 6673 (72·7%) women who provided at least two cervical cell samples (appendix p 5). Persistent HPV 16/18 infection was observed in seven (0·1%; 95% CI 0·0–0·2) of 6673 vaccinated women, with similar frequencies observed between the single-dose cohort and other dose cohorts (table 2). A single case of persistent HPV 16 infection was observed among the single-dose recipients. There was no case of persistent HPV 18 infection in this group. Unvaccinated women had a higher frequency of persistent HPV 16/18 infections (32 [2·5%; 95% CI 1·7–3·6] of 1265) than the vaccinated women (seven [0·1%; 0·0–0·2] of 6673; table 2). Persistent infection from the cross-protective HPV types was less frequent in vaccinated women than in unvaccinated women. Similar frequencies of persistent infection from cross-protective types was observed among the dose groups (table 2).

Table 3 presents the effect of participant characteristics on the frequencies of other non-vaccine-targeted HPV types excluding 31, 33, and 45 among unvaccinated women. In the adjusted analysis from the logistic regression model, background HPV infection frequency, time between date of marriage and first cervical specimen collection, and number of cervical specimens per participant were the only independent predictors of incidence of other non-vaccine-targeted HPV types excluding 31, 33 and 45. Other assessed factors of birth cohort, religion, total number of pregnancies, ages at first cervical cell sample collection, and delay to cervical sample collection were not associated with incidence of other non-vaccine targeted HPV types excluding 31, 33 and 45. Thus, these three factors (background HPV infection frequency, time between date of marriage and first cervical specimen collection, and number of cervical specimens per participant) were used to fit the participant disease risk scores, which in turn were used to form the five strata for the assessment of vaccine efficacy.

**Table 3:** Distribution and effect of women's characteristics on the incidence of infections with non-vaccine-targeted HPV types excluding 31, 33, and 45 among unvaccinated women (first unvaccinated cohort)

		**Women assessed**	**Women positive**	**Percentage positive (95% CI)**	**Crude odds ratio (95% CI)**	Adjusted*odds ratio (95% CI)
Overall		1484	403	27·2% (24·9–29·5)	..	..
Background HPV infection rate status†						
	Low	738	195	26·4% (23·3–29·8)	1 (ref)	1 (ref)
	Medium	646	152	23·5% (20·3–27·0)	0·9 (0·7–1·1)	1·1 (0·8–1·4)
	High	100	56	56·0% (45·7–65·9)	3·5 (2·3–5·4)	2·6 (1·2–5·4)
Birth cohort						
	<1995	1316	355	27·0% (24·6–29·5)	1 (ref)	1 (ref)
	≥1995	168	48	28·6% (21·9–36·0)	1·1 (0·8–1·5)	1·1 (0·8–1·7)
Religion						
	Hindu	1298	318	24·5% (22·2–26·9)	1 (ref)	1 (ref)
	Others	186	85	45·7% (38·4–53·1)	2·6 (1·9–3·6)	1·4 (0·8–2·2)
Total number of pregnancies						
	None	182	68	37·4% (30·3–44·8)	1 (ref)	1 (ref)
	One	510	153	30·0% (26·1–34·2)	0·7 (0·5–1·0)	1·3 (0·8–2·1)
	Two or more	792	182	23·0% (20·1–26·1)	0·5 (0·4–0·7)	0·9 (0·5–1·4)
Age at first cervical cell sample collection, years						
	<21	873	252	28·9% (25·9–32·0)	1 (ref)	1 (ref)
	≥21	611	151	24·7% (21·3–28·3)	0·8 (0·6–1·0)	0·9 (0·7–1·2)
Time between dates of marriage and first cervical sample collection, years						
	<2	391	129	33·0% (28·3–37·9)	1 (ref)	1 (ref)
	2 to <3	392	90	23·0% (18·9–27·4)	0·6 (0·4–0·8)	0·7 (0·5–0·9)
	≥3	696	181	26·0% (22·8–29·4)	0·7 (0·5–0·9)	0·9 (0·6–1·2)
Delayed cervical sample collection‡						
	None delayed	370	76	20·5% (16·5–25·0)	1 (ref)	1 (ref)
	At least one delayed	1114	327	29·4% (26·7–32·1)	1·6 (1·2–2·1)	0·8 (0·5–1·2)
Number of cervical cell samples per participant						
	One to two	403	66	16·4% (12·9–20·4)	1 (ref)	1 (ref)
	Three or more	1081	337	31·2% (28·4–34·0)	2·3 (1·7–3·1)	2·9 (1·9–4·6)

HPV=human papillomavirus. HPV types 26, 35, 39, 51, 52, 53, 56, 58, 59, 66, 68, 70, 73, and 82 were included.

* Adjusted for all characteristics.

† Three categories (low rates: <10%; medium rates: 10 to <16%; and high rates: ≥16%) of the site-specific and HPV vaccination status-specific background HPV rates of the non-vaccine-targeted types, excluding 31, 33, and 45, using only the participants' first cervical cell sample collections.

‡ A participant was defined as having a delayed sample collection date if she had gap of 18 months or more between any consecutive sample collection dates. A participant who had less than four consecutive sample collections and whose time between the latest date sample collection overall and her last sample collection was more than 18 months was also defined as having a delayed sample collection. All other participants not fulfilling the above two criteria were defined as not having delayed sample collection.

Since there were very few records with missing data (five missing dates of marriage for the unvaccinated group), they were excluded from the vaccine efficacy analysis. Adjusted vaccine efficacy against incident HPV 16/18 infection in the single-dose recipients was 63·5% (95% CI 51·2–73·1), which was similar to the vaccine efficacies observed in the two-dose cohort (67·7%; 55·2–77·2) and three-dose cohort (66·4%; 53·6–76·3; table 4). There was no significant difference between the dose groups in vaccine efficacy against incident infection from either the cross-protective HPV types or the 21 HPV types combined.

**Table 4:** Vaccine efficacy for the prevention of incident and persistent HPV infections

		**Unvaccinated cohort**	**Single-dose default cohort**	**Two-dose cohort**	**Three-dose cohort**
**Incident HPV**					
Women assessed		1479	2858	2166	2019
Incident HPV 16 and 18 infections					
	Observed events	138	92	59	59
	Crude attack rates	9·33%	3·22%	2·72%	2·92%
	Adjusted vaccine efficacy* (95% CI)	..	63·5% (51·2 to 73·1)	67·7% (55·2 to 77·2)	66·4% (53·6 to 76·3)
	Difference in vaccine efficacy† (95% CI)	..	..	4·2% (−7·1 to 16·0)	3·0% (−9·1 to 14·8)
Incident HPV 16, 18, 6, and 11 infections					
	Observed events	192	154	107	110
	Crude attack rates	12·98%	5·39%	4·94%	5·45%
	Adjusted vaccine efficacy* (95% CI)	..	54·1% (41·8 to 64·1)	59·0% (46·9 to 69·1)	54·7% (40·9 to 65·0)
	Difference in vaccine efficacy† (95% CI)	..	..	4·8% (−6·0 to 16·1)	0·6% (−11·2 to 11·9)
Incident HPV types 31, 33 and 45 infections					
	Observed events	148	136	89	86
	Crude attack rates	10·01%	4·76%	4·11%	4·26%
	Adjusted vaccine efficacy* (95% CI)	..	43·5% (25·4 to 56·5)	54·0% (38·5 to 66·5)	54·6% (38·3 to 66·6)
	Difference in vaccine efficacy† (95% CI)	..	..	10·6% (−3·8 to 24·6)	11·1% (−2·9 to 25·7)
Any incident HPV infection					
	Observed events	557	667	493	496
	Crude attack rates	37·66%	23·34%	22·76%	24·57%
Adjusted vaccine efficacy* (95% CI)		..	30·2% (20·1 to 38·5)	34·5% (24·5 to 43·1)	30·2% (19·7 to 39·4)
Difference in vaccine efficacy† (95% CI)		..	..	4·3% (−4·1 to 12·1)	−0·1% (−8·3 to 8·5)
**Persistent HPV**					
Women assessed		1260	2135	1452	1460
Persistent HPV 16 and 18 infections					
	Observed events	32	1	1	1
	Crude attack rates	2·54%	0·05%	0·07%	0·07%
	Adjusted vaccine efficacy* (95% CI)	..	95·4% (85·0 to 99·9)	93·1% (77·3 to 99·8)	93·3% (77·5 to 99·7)
	Difference in vaccine efficacy† (95% CI)	..	..	−2·0% (−20·2 to 11·3)	−1·9% (−19·4 to 12·4)
Persistent HPV 16, 18, 6, and 11 infections					
	Observed events	35	2	1	2
	Crude attack rates	2·78%	0·09%	0·07%	0·14%
	Adjusted vaccine efficacy* (95% CI)	..	93·4% (81·1 to 99·1)	93·7% (79·8 to 99·8)	90·3% (71·9 to 98·5)
	Difference in vaccine efficacy† (95% CI)	..	..	0·3% (−16·6 to 14·5)	−2·8% (−21·6 to 12·6)
Persistent HPV types 31, 33, and 45 infections					
	Observed events	14	14	11	7
	Crude attack rates	1·11%	0·66%	0·76%	0·48%
	Adjusted vaccine efficacy* (95% CI)	..	8·8% (−230·8 to 62·6)	8·4% (−239·3 to 65·7)	38·8% (−124·4 to 80·2)
	Difference in vaccine efficacy† (95% CI)	..	..	0·0% (−104·0 to 101·8)	27·9% (−51·9 to 138·6)
Any persistent HPV infection					
	Observed events	100	80	55	55
	Crude attack rates	7·94%	3·75%	3·79%	3·77%
	Adjusted vaccine efficacy* (95% CI)	..	35·4% (3·7 to 56·0)	36·7% (1·6 to 57·9)	39·3% (6·8 to 60·2)
	Difference in vaccine efficacy (95% CI)	..	..	1·2% (−22·0 to 24·2)	3·7% (−18·9 to 26·9)

HPV=human papillomavirus.

* Adjusted through direct standardisation on the five strata created from the disease risk score estimates.

† Alternative dose minus the single dose.

The adjusted vaccine efficacy of a single dose against persistent HPV 16/18 infection was 95·4% (95% CI 85·0–99·9), which was not significantly different from that observed with two doses (93·1%; 77·3–99·8) and three doses (93·3%; 77·5–99·7; table 4). Efficacy against persistent infection from all HPV types was 35·4% (95% CI 3·7–56·0) for single-dose recipients, 36·7% (1·6–57·9) for two-dose recipients, and 39·3% (6·8–60·2) for three-dose recipients.

The HPV screening test was positive in 197 (4·1%) of the 4819 vaccinated and 277 (6·0%) of the 4626 unvaccinated women (appendix p 6). HPV 16/18 (using the *digene* PS test) was detected in one (0·1%) of 1037 three-dose recipients, four (0·3%) of 1143 the two-dose recipients, two (0·1%) of 1511 single-dose recipients, and 63 (1·4%) of 4626 the unvaccinated women. The single case of CIN3 detected in the vaccinated women was a single-dose recipient. She was negative for HPV 16/18, both on the Luminex assay and *digene* PS test. No CIN2 or invasive cancer was detected in the vaccinated cohorts. Five cases of CIN2 or CIN3 were detected among the unvaccinated women, three of which were associated with HPV 16/18. One case of invasive cancer, not associated with HPV 16/18, was detected in the unvaccinated women cohort.

## Discussion

Based on systematic follow-up of more than 4000 adolescent girls receiving a single dose, our study has demonstrated very high efficacy of one dose of the quadrivalent vaccine against persistent infection from HPV 16/18, which is sustained until 10 years post vaccination. Vaccine efficacy of one dose was no different from that observed for two or three doses of the same vaccine.

An expert group convened by the IARC in 2013 unanimously decided that demonstration of protection against persistent cervical infection would be considered adequate proof of efficacy of a single dose of HPV vaccine to prevent advanced cervical precancers or cancer.^15^ Our study provides evidence to support that a single dose of HPV vaccine fulfils the conditions set out by the IARC expert group to be considered as protective against cervical cancer caused by HPV 16/18—the types responsible for at least 70% of cervical cancers detected globally.^16^

High efficacy of single dose of a HPV vaccine against persistent HPV 16/18 infection was previously reported by a combined post-hoc analysis of the Costa Rica Vaccine trial and PATRICIA trial in a smaller number of single-dose recipients (n=292).^17^ Durability of protection offered by the HPV vaccine is a major concern from public health viewpoint because the vaccinated girls will remain at risk of being infected for decades. The antibody concentrations in single-dose recipients stabilise 18–24 months after vaccination and remain sustained at a level higher than that observed after natural infection for at least a decade, thus explaining the durable protection observed in our study.^18^, ^19^ A single dose providing a strong and sustained protection could be biologically explained by the fact that the repetitive epitopes on the virus-like particles promote efficient induction of the long-lived plasma cells through multivalent engagement of the B-cell receptors and that a remarkably low titre of antibody can effectively neutralise the virus.^20^, ^21^ Unsurprisingly, several countries introducing the HPV vaccine into their national immunisation programmes early have demonstrated high efficacy of a single dose similar to that of higher number of doses against vaccine-targeted HPV infections, anogenital warts, and histopathology-proved CIN2+ lesions.^22^, ^23^, ^24^, ^25^, ^26^

The non-randomised design of our study could be considered as a limitation. However, the fact that allocation of the participants to different dose groups happened by default and was not controlled by the investigators or decided on by the participants reduces any selection bias. A recent systematic review of single-dose efficacy trials accepted that our study had low risk of exposure or outcome misclassification, since the dose cohorts were well matched for age and sociodemographic characteristics and were followed up in the same manner.^27^ We accept that, due to the non-randomised design, there might be residual confounding circumstances, such as geographical, ethnicity, and cultural factors, which we might not have been able to adjust for. The higher frequency of non-vaccine-targeted HPV infection observed in our unvaccinated cohort than in the vaccinated cohorts indicates an imbalance in the risk of infection between the two. However, this somewhat imperfect selection of the unvaccinated controls is negated by the fact that single-dose recipients had similar vaccine efficacy to the two-dose and three-dose recipients. Any conceivable bias resulting from higher risk of HPV infections in the unvaccinated comparator is likely to affect the vaccine efficacy estimates in all three groups equally. The proportion of participants eligible to provide samples for genotyping was different across the dose groups. However, the proportion of eligible participants assessed for incident and persistent infections in each group was similar. The large sample size, systematic follow-up of the participants over many years, blinded assessment of outcomes, and use of two independent HPV detection tests are the major strengths of our study. We believe that documentation of only a single case of persistent HPV 16 infection among 2136 single-dose recipients strongly supports the high efficacy of a single dose. The results of our study are generalisable as our single-dose cohort comprising sexually uninitiated, average-risk adolescent girls is representative of the primary target population for HPV vaccination. However, our results are not applicable for immunocompromised girls.

Nearly half of LMICs have not been able to introduce HPV vaccination because most of them have phased out of support from Gavi, the Vaccine Alliance, and many countries cannot afford the price of US$4·50 per dose negotiated by Gavi.^28^ The health crisis created by COVID-19 pandemic and the channelling of resources to procure vaccines against SARS-CoV-2 will have a disruptive effect on the under-funded cancer control programmes in LMICs.^29^ Global elimination of cervical cancer will be achievable only when this huge inequity is addressed with pragmatism. A preprint modelling study has shown that a single dose of HPV vaccine even with somewhat lower efficacy, but with higher coverage can have greater population-level effect than two doses.^30^ Herd immunity created in the population through high coverage could potentially compensate for any waning protection of a vaccine over time at an individual level.^15^ A judicious recommendation to introduce a single dose of HPV vaccine and ensure high coverage of HPV vaccination will contribute to elimination of cervical cancer in a more affordable manner.

## Data sharing

External researchers can make written requests to the IARC for sharing of data after publication. Requests will be assessed on a case-by-case basis in consultation with lead and co-investigators. A brief analysis plan and data request will be required and reviewed by the investigators for approval of data sharing. When requests are approved, anonymised data will be sent electronically in password protected files. All data sharing will abide by rules and policies defined by the sponsor; relevant institutional review boards; local, state, and federal laws and regulations. Data sharing mechanisms will ensure that the rights and privacy of individuals participating in research will be protected at all times.

## Declaration of interests

PB has received research funding from GlaxoSmithKline through the Chittaranjan National Cancer Institute (Kolkata, India) during his previous position at the institute. NB has received research funding through her institute from GlaxoSmithKline and Merck. SJ has received funds from GlaxoSmithKline through the Jehangir Clinical Development Center (Pune, India) for a human papillomavirus vaccine study. All other authors declare no competing interests.
