# Correction to *Lancet Oncol* 2021; 22: 1518–29

**DOI:** 10.1016/S1470-2045(21)00700-2

**Published:** 2022-01

*Basu P, Malvi SG, Joshi S, et al. Vaccine efficacy against persistent human papillomavirus (HPV) 16/18 infection at 10 years after one, two, and three doses of quadrivalent HPV vaccine in girls in India: a multicentre, prospective, cohort study.* Lancet Oncol *2021;* 22: *1518–29*—In figure 2 of this Article, the numbers at risk and censored for year 5 were omitted and the x-axis label should have been “Time since marriage (years)”. These corrections have been made to the online version as of Dec 29, 2021.

