# Supplementary material for: Vaccine efficacy against persistent human papillomavirus (HPV) 16/18 infection at 10 years after one, two, and three doses of quadrivalent HPV vaccine in girls in India: a multicentre, prospective, cohort study
Source: Lancet Oncol. 2021 Nov;22(11):1518–29. doi: 10.1016/S1470-2045(21)00453-8 (PMC8560643; doi:10.1016/S1470-2045(21)00453-8)
Supplement: Supplementary appendix [file mmc1.pdf]

THE LANCET
Oncology

Supplementary appendix

This appendix formed part of the original submission and has been peer reviewed. We post it as supplied by the authors.

Supplement to: Basu P, G Malvi S, Joshi S, et al. Vaccine efficacy against persistent human papillomavirus (HPV) 16/18 infection at 10 years after one, two, and three doses of quadrivalent HPV vaccine in girls in India: a multicentre, prospective, cohort study. *Lancet Oncol* 2021; published online Oct 8. http://dx.doi.org/10.1016/S1470-2045(21)00453-8.

Appendix and Supplementary materials

Forming strata

The formation of the strata included the following steps. First, logistic regression models were performed to assess whether those other non-vaccine targeted HPV types significantly determine the vaccine targeted (HPV 16 and/or 18) and cross-protective (HPV 31, 33 and/or 45) outcomes (Supplementary table 1). This was shown. Second, using only the unvaccinated cohort, women characteristics were put in a logistic regression model to assess their effect on the other non-vaccine targeted HPV types excluding 31, 33 and 45 (the infections that are unlikely to be affected by vaccination). These HPV types were used to mimic infections rates in the absence of vaccination. The participant characteristics included in the logistic regression model were, background site-specific HPV infection prevalence status (<10% - low, 10-<16%-medium and ≥16% - high) of the non-vaccine targeted types, excluding 31,33 and 45 among the unvaccinated, estimated using only the participants' first cervical samples; birth cohort (<1995 and 1995+); religion (Hindu and others); participant number of pregnancies (none, one and two or more); age at first cervical cell sample collection (<21 and ≥21 years); time between date of marriage and first cervical sample collection (<2, 2 to <3 and ≥3 years); delayed yearly cervical sample collection (none delayed and at least one delayed beyond the window period of six months); and number of cervical cell samples per participant (1-2 and ≥3). Supplementary table 5 shows the HPV infections rates obtained using only data of the participants' first cervical cell samples collected. These rates were used as a proxy to assess the baseline site-specific HPV infection profile. The attack rates in the unvaccinated women were used to create 3 categories of the background HPV infection rates (<10% - low, 10-<16%-medium and ≥16% - high). Since the site-specific rates were similar between the vaccinated and unvaccinated groups, and since Mumbai site did not have women recruited in the unvaccinated group, we used the estimated rate in the vaccinated group in place of the unvaccinated HPV background rate. A participant was defined as having a delayed sample collection date if she had gap of 18 months or more between any consecutive sample collections dates. A participant who had less than four consecutive sample collections and whose time between the latest date sample collection overall and her last sample collection was more than 18 months was also defined as having a delayed sample collection. Third, was the estimation of each participant's disease risk score using the variables in the regression model that were significantly influencing these other HPV types at 5% significance level following the procedure described by Sullivan et al.[a] The significant variables were further run in another logistic regression model (again using only unvaccinated cohort and the other non-vaccine targeted HPV types excluding 31, 33 and 45 as the outcome) to obtain the coefficients used in the procedure. We calculated the disease risk scores based on the unvaccinated group to minimize the bias in case the vaccine-dose received was correlated with the selected variables above. Fourth, we then created five strata from the fitted scores using the lowest level as the first stratum of individuals with minimal or no risk and the remaining four from quantiles of the scores. As such, we avoided assuming that the risk score and the outcome were related through a particular function.[b]

The following three factors were used in the estimation of disease risk scores, which in turn were used to create 5 analysis strata

1. Because of the varying site-specific rates, site- and HPV vaccination status-specific background HPV attack rates of the non-vaccine targeted types, excluding 31,33 and 45 were calculated using only the participant first cervical cell sample collections. The following 3 categories of these background rates were then created.
   - Low rates (<10%)
   - Medium rates (10-<16%)
   - High rates (≥16%)
2. Three categories of time period between dates of marriage and first cervical sample collection:
   - <2 years
   - 2 to <3 years
   - 3 years or more

3. Two categories of the number of cervical samples collections:
   - 1-2 samples for the analysis of incident infections or 2 samples for the analysis of persistent infections
   - >2 samples

References

a. Sullivan LM, Massaro JM, D'Agostino RB Sr. Presentation of multivariate data for clinical use: The Framingham Study risk score functions. Stat Med. 2004 May 30;23(10):1631-60.

b. Arbogast PG, Kaltenbach L, Ding H, Ray WA. Adjustment for multiple cardiovascular risk factors using a summary risk score. Epidemiology. 2008 Jan;19(1):30-7.

Supplementary table 1: Prediction of the Incident HPV 16 and/or 18 and incident HPV 31, 33 and/or 45 by the other non-vaccine targeted HPV types excluding 31, 33 and 45

**Non-vaccine targeted HPV types excluding 31, 33 and/or 45**	**Women assessed n**	**Positive for the particular outcome n (%)**	**Crude odds ratio (95% CI)**	**p-value**	**Adjusted* odds ratio (95% CI)**	**p-value**
			Incidence HPV 16 and/or 18 outcome			
Negative	1,081	55 (5·1)	1·00		1·00	
Positive	403	85 (21·1)	4·99 (3·47 - 7·16)	<0·001	3·57 (2·41 - 5·28)	<0·001
Total	1,484	140 (9·4)				
			Incidence HPV 31,33 and/or 45 outcome			
Negative	1,081	74 (6·8)	1·00		1·00	
Positive	403	74 (18·4)	3·06 (2·17 - 4·32)	<0·001	2·50 (1·72 - 3·64)	<0·001
Total	1,484	148 (10·0)				

HPV: human papillomavirus; CI: confidence interval; * Adjusted for all women characteristics (study site, birth cohort, religion, education, time between dates of marriage and first cervical sample collection, delayed cervical sample collection and number of cervical cell sample collections per participant

The other important consideration was to see to what extent the non-vaccine targeted HPV types excluding 31, 33 and 45 predict types 16 and/or 18, t and types 31, 33, and/or 45. As demonstrated in Table 4, all the three outcomes were predicated by the non-vaccine targeted HPV types excluding 31, 33 and 45.

Supplementary table 2: Distribution of participants with at least one cervical sample collection for HPV genotyping by study site and dose received

Study site	3-dose (Days 1, 60 and ≥180) n (%)		2-dose (Days 1 and ≥180) n (%)		2 dose default (Days 1 and 60) n (%)		A single dose n (%)		First* unvaccinated cohort n (%)	
Ambillikai, Tamil Nadu	506	(25·1)	647	(29·9)	61	(2·9)	124	(4·3)	200	(13·5)
Barshi, Maharashtra	493	(24·4)	554	(25·6)	1757	(82·1)	1880	(65·8)	188	(12·7)
New Delhi, Delhi	127	(6·3)	132	(6·1)	20	(0·9)	18	(0·6)	200	(13·5)
Ahmedabad, Gujarat	0	(0·0)	0	(0·0)	0	(0·0)	433	(15·2)	50	(3·4)
Hyderabad, Telangana	0	(0·0)	0	(0·0)	108	(5·0)	140	(4·9)	246	(16·6)
Mumbai, Maharashtra	0	(0·0)	53	(2·4)	0	(0·0)	6	(0·2)	0	
Pune, Maharashtra	803	(39·8)	703	(32·5)	188	(8·8)	244	(8·5)	400	(27·0)
Gangtok, Sikkim	34	(1·7)	24	(1·1)	3	(0·1)	8	(0·3)	100	(6·7)
Aizawl, Mizoram	56	(2·8)	53	(2·4)	3	(0·1)	5	(0·2)	100	(6·7)
Total	2019	(100·0)	2166	(100·0)	2140	(100·0)	2858	(100·0)	1484	(100·0)
Median age of participants at the time of first cervical sample collection (years, IQR)	21	(19-22)	21	(20-23)	20	(19-22)	21	(19-22)	20	(19-21)

*Site distribution of the participants recruited to the second unvaccinated cohort (screening only cohort) is shown in the supplementary table 2, since no cervical sample was collected from them for HPV genotyping

Supplementary table 3: Participant eligibility and provision of cervical specimen collections for HPV genotyping (Luminex™ assay) and/or participation in cervical cancer screening using Hybrid Capture II™ test

	Participants vaccinated	Cervical specimen collection for HPV genotyping (Luminex™ assay)						Screening for cervical cancer using Hybrid Capture II™ (HC II)			
		Participants eligible[a]		Participants with at least one collection for HPV incidence analysis		Participants with at least two collections for HPV persistence analysis		Participants eligible[b]		Participants screened with HC II	
	n	n (%)[c]		n (%)[d]		n (%)[e]		n (%)[c]		n (%)[d]	
All participants	17,729	10,915	(61·6)	9,183	(84·1)	6,673	(72·7)	7,091	(40·0)	4,819	(68·0)
Vaccine dose received											
3-dose (days 1, 60 and ≥180)	4,348	2,416	(55·6)	2,019	(83·6)	1,460	(72·3)	1,540	(35·4)	1,037	(67·3)
2-dose (days 1 and ≥180)	4,980	2,598	(52·2)	2,166	(83·4)	1,452	(67·0)	1,734	(34·8)	1,143	(65·9)
2-dose default (days 1 and 60)	3,452	2,491	(72·2)	2,140	(85·9)	1,626	(76·0)	1,622	(47·0)	1,128	(69·5)
A single dose	4,949	3,410	(68·9)	2,858	(83·8)	2,135	(74·7)	2,195	(44·4)	1,511	(68·8)
Study site											
Ambillikai	3,300	1,699	(51·5)	1,338	(78·8)	783	(58·5)	1,153	(34·9)	646	(56·0)
Barshi	7,092	5,418	(76·4)	4,684	(86·5)	3,632	(77·5)	3,454	(48·7)	2,527	(73·2)
Delhi	1,000	373	(37·3)	297	(79·6)	202	(68·0)	286	(28·6)	199	(69·6)
Ahmedabad	1,011	570	(56·4)	433	(76·0)	278	(64·2)	292	(28·9)	170	(58·2)
Hyderabad	794	347	(43·7)	248	(71·5)	168	(67·7)	212	(26·7)	98	(46·2)
Mumbai	514	78	(15·2)	59	(75·6)	30	(50·8)	61	(11·9)	38	(62·3)
Pune	3,018	2,179	(72·2)	1,938	(88·9)	1,433	(73·9)	1,441	(47·7)	1,021	(70·9)
Sikkim	500	112	(22·4)	69	(61·6)	60	(87·0)	92	(18·4)	42	(45·7)
Mizoram	500	139	(27·8)	117	(84·2)	87	(74·4)	100	(20·0)	78	(78·0)

[a] starting at 18 months after marriage or 6 months after first pregnancy, whichever is earlier; [b] married participants when they reach 25 years of age; [c] proportion obtained using participants vaccinated as denominator; [d] proportion obtained using participants eligible as denominator; [e] proportion obtained using participants with at least one collection for HPV incidence analysis as denominator

Supplementary table 4: Hybrid capture II test positivity and CIN detection by HPV vaccine dose received

	Women screened with HCII	Women screened positive				CIN detection				CIN associated with HPV types 16 and/or 18			
		Overall		For HPV 16 and/or 18		CIN 1	CIN 2	CIN 3	Invasive cancer	CIN 1	CIN 2	CIN 3	Invasive cancer
	n	n (%)		n (%)		n	n	n		n	n	n	n
Unvaccinated group	4,626	277	(6·0)	63	(1·4)	16	4	1	1	5	2	1	0
Vaccinated group	4,819	197	(4·1)	7	(0·1)	12	0	1	0	1	0	0	0
3-dose (Days 1, 60 and ≥180)	1,037	46	(4·4)	1	(0·1)	2	0	0	0	0	0	0	0
2-dose (Days 1 and ≥180)	1,143	61	(5·3)	4	(0·3)	5	0	0	0	1	0	0	0
2 doses (Days 1 and 60)	1,128	31	(2·7)	0	(0·0)	1	0	0	0	0	0	0	0
A single dose	1,511	59	(3·9)	2	(0·1)	4	0	1	0	0	0	0	0

HCII: hybrid capture II; HPV: human papillomavirus; CIN: cervical intraepithelial neoplasia

Supplementary table 5: Positivity for non-vaccine targeted HPV types excluding HPV 31,33 and 45 by study sites and vaccination status

Site	Vaccinated group*		Unvaccinated group	
	Women assessed	Positive for the non- vaccine targeted HPV types excluding 31, 33 and 45	Women assessed	Positive for the non- vaccine targeted HPV types excluding 31, 33 and 45
	n	n (%)	n	n (%)
All	7043	683 (9·7)	1484	172 (11·6)
Site				
Ambillikai	1277	160 (12·5)	200	25 (12·5)
Barshi	2927	226 (7·7)	188	15 (8·0)
New Delhi	277	27 (9·7)	200	21 (10·5)
Ahmedabad	433	51 (11·8)	50	3 (6·0)
Hyderabad	140	18 (12·9)	246	34 (13·8)
Mumbai	59	11 (18·6)	0	
Pune	1750	138 (7·9)	400	35 (8·8)
Sikkim	66	12 (18·2)	100	8 (8·0)
Mizoram	114	40 (35·1)	100	31 (31·0)

HPV: human papillomavirus; * Includes women who received either a single-dose, 2 doses (Days 1 and 180+) or 3 doses (Days 1, 60 and 180+)

Supplementary table 6: List of study sites, site principal investigators and total number of participants recruited

Name of study site	Principal investigator responsible for the site	Number of participants recuited at the site
Barshi, Maharashtra	Sylla G. Malvi	8843
Ambillikai, Tamilnadu	Pulikottil O. Esmy	4100
Pune, Maharashtra	Smita Joshi	4018
New Delhi, Delhi	Neerja Bhatla	1500
Hyderabad, Telangana	Usha Rani Reddy Poli	1361
Ahmedabad, Gujarat	Anand Shah	1161
Gangtok, Sikkim	Yogesh Verma	704
Aizawl, Mizoram	Eric Zomawia	700
Mumbai, Maharashtra	Sharmila Pimple	514
